# Examining determinants of stunting in Urban and Rural Indonesian: a multilevel analysis using the population-based Indonesian family life survey (IFLS)

**DOI:** 10.1186/s12889-024-18824-z

**Published:** 2024-05-22

**Authors:** Issara Siramaneerat, Erni Astutik, Farid Agushybana, Pimnapat Bhumkittipich, Wanjai Lamprom

**Affiliations:** 1 Department of Social Science, Faculty of Liberal Arts, Rajamangala University of Technology Thanyaburi (RMUTT), 39 Moo1, Klong 6, Khlong Luang, Pathum Thani, 12110 Thailand; 2 Department of Epidemiology, Population Biostatistics and Health Promotion, Faculty of Public Health, Airlangga University, JI. Mulyorejo, Surabaya, Jawa Timur, 60115 Indonesia; 3 Department of Biostatistics and Demography, Faculty of Public Health, Diponegoro University, Jl. Prof. Soedarto, SH. Tembalang, Semarang, Central Java, 50275 Indonesia

**Keywords:** Stunting, Malnutrition, Undernutrition, Indonesia, IFLS

## Abstract

**Background:**

In Indonesia, chronic malnutrition leading to stunted growth in children represents a significant issue within the public health domain. The prevalence of stunting varies between urban and rural areas, reflecting disparities in access to nutrition, healthcare, and other socioeconomic factors. Understanding these disparities is crucial for developing targeted interventions to address the issue.

**Methods:**

The study used data from the fifth wave of the Indonesian Family Life Survey (IFLS), which is a national cross-sectional population-based survey conducted across approximately 13 provinces in Indonesia in 2014–2015. Multivariate and Multilevel logistic regression models were utilized in the analysis to determine the factors associated with the prevalence of stunting in Indonesian children.

**Results:**

The multivariate logistic regression analysis indicated that among children aged 24–59 months in Indonesia, stunting was associated with the age of the child, birth weight, maternal nutritional status, and residence. Subsequently, the multilevel logistic regression analysis revealed that in rural areas, the age of the child and birth weight exhibited significant associations with stunting. Conversely, in urban areas, stunted children were influenced by 7 factors, including the child’s age (months), age of weaning, birth weight (kg), mother and father’s age, place of birth, and maternal nutritional status.

**Conclusions:**

Variations in childhood stunting between urban and rural regions in Indonesia were observed, indicating a differential prevalence. The study’s findings suggests the importance of age-appropriate nutritional support, healthcare interventions, and growth monitoring. Focused interventions are vital, potentially encompassing initiatives such as improving access to maternal and child healthcare services, promoting adequate nutrition during pregnancy and infancy, and facilitate greater parental engagement in childcare responsibilities.

## Introduction

Malnutrition continues to be a significant global nutritional issue, remaining the primary cause of death in children under the age of 5 worldwide. Children afflicted with malnutrition undergo delayed growth and development compared to their peers of similar age. They are often thin and underweight, which puts them at risk of developing infections and other health problems [1]. The International Food Policy Research Institute (IFPRI) report suggests that more comprehensive solutions are needed to address these issues, including improved access to nutritious foods, better healthcare, and increased education on healthy eating habits [2, 3].

Indonesia faces a significant challenge of malnutrition, particularly in the form of stunting. Despite being one of the highest food-producing countries [4], data from the Ministry of Health reveals that 30% of children with growth impairment and 92% of the population consume quantities of fruits and vegetables far below the limits set by the World Health Organization (WHO) [5]. Therefore, tackling child stunting remains a key government commitment as outlined in the Indonesia Medium Development Goals 2020–2024 [6].

A review of the literature found that underlying factors at the child, parent, and household levels were associated with stunting [7–9]. Moreover, studies have demonstrated that children raised by educated parents are at a lower risk of stunting. Parental education plays a significant role in reducing the risk of stunting, as educated parents are more likely to provide care in the form of immunizations, vitamin A, and iodized salt for their children, thereby reducing the risk of stunting [10]. Additionally, children’s eating habits and nutritional status are influenced by parental characteristics and the household’s socioeconomic background [11].

Importantly, studies showed that the disparity between urban and rural areas in the prevalence of stunting among children can vary. Research in Tanzania indicated that although the overall prevalence of stunting in children under five has significantly decreased over the past three decades, the burden of stunting among children in rural areas remains high [7]. Another study suggested differences in the significant reduction rates of stunting between children living in urban and rural areas [8]. Additionally, in more advanced urban communities, the likelihood of stunting was lower than in rural communities due to the presence of antenatal care, health care centers, and comprehensive nutrition [8–10]. The study suggested that low birth weight and parental height are common determinants of stunting across different areas, other factors such as sanitation, income of the household, and maternal education play a significant role and can have different impacts in urban versus rural contexts [11]. In the contract, a further study from Indonesia [12] analyzed risk factors of mild and severe stunting in both rural and urban areas of Indonesia. It was found that low birth weight, and low to middle economic status were strong determinant factors for stunting and severe stunting in both urban and rural.

This study explores the difference in stunting prevalence between urban and rural areas among Indonesian children aged 24–59 months, using data from the 2014 Indonesia Family Life Survey (IFLS). Focusing on children in this age group rather than provides valuable insights into the lasting effects of early nutritional deficiencies and other factors contributing to stunting. Understanding these determinants in the 24–59 months age group helps identify factors that can be changed and informs targeted interventions to prevent ongoing stunting and encourage catch-up growth. Limited research has explored the multi-level factors of children, parents, and households comparing the outcomes between urban and rural residents. Therefore, investigating the disparities in stunting among Indonesian children in urban and rural areas is vital for developing evidence-based interventions, addressing health disparities, and enhancing the well-being of children across different geographic and socioeconomic contexts.

## Methods

### Study design and participants

This cross-sectional population-based national data was obtained from the fifth wave of the Indonesian Family Life Survey (IFLS) conducted in 2014–2015. IFLS-5 used a multistage stratified sampling design that represents 83% of the Indonesian population and was conducted in 13 provinces in Indonesia. These provinces include North Sumatra, Yogyakarta, West Sumatra, East Java, South Sumatra, Bali, Lampung, West Nusa Tenggara, Jakarta, South Kalimantan, West Java, South Sulawesi, and Central Java [13]. The 13 provinces were chosen with regard to Indonesia’s cultural and socioeconomic diversity, as well as to maximize population representation. A nationally representative sample frame was utilized to randomly choose 321 enumeration areas (EA) within each of the 13 provinces. Households in each EA were selected at randomly using the 1993 Survei Sosial Ekomomi Nasional (SUSENAS) listings that were acquired from the local Badan Pusat Statistic (BPS) office. Ultimately, 20 households were selected from each urban EA, and 30 households were selected from each rural EA. The data from this study is available online at https://www.rand.org/well-being/social-and-behavioral-policy/data/FLS/IFLS.html. This study focused on families with children aged 24–59 months. After removing the missing cases, the total number of samples 5 is 2,428 [14].

Moreover, the IFLS5 surveys and their procedures were properly reviewed and approved by Institutional Review Boards (IRBs) of RAND Corporation in the United States and in Indonesia at the University of Gadjah Mada (UGM). The data collected from the IFLS surveys aims to collect data on various aspects of Indonesian households, including demographics, health, education, and income to inform the policy decisions and measure progress toward development goals in Indonesia [13].

### Data management

The outcome variable in this study was the stunting status of children aged 24–59 months. The stunting variable was divided into two categories: stunting (height less than the median by more than two standard deviations) and normal or height (height equal to or greater than the median by more than two standard deviations). In this study, “1” is assigned to indicate stunted growth in the child, while “0” indicates no stunting. This outcome variable was measured using the Z-Score calculation of the child’s height and age based on the child’s gender. Height and Body mass index (BMI) z-scores were calculated based on the 2006 WHO child growth standards for children less than 5 years of age [15]. Furthermore, the variables used in this study were developed based on the IFLS handbook, namely Book US, Book 1, 2, 3 A, 4, and 5.

### Individual-level variables

Child factors include child’s age in months, child’s gender (female (1) and male (0)), birth weight (less than 2.5 kg (0), 2.5–3.99 kg (1), and more than 4 kg (2)), age of weaning (less than 6 months (1) and more than 6 months (0)), The timing of introduction of complementary feeding (less than 6 months (1) and more than 6 months (0)), and The timing of introduction of water (less than 6 months (1) and more than 6 months (0)). The data on the age child and the gender of the child are taken from Book 5. The variables of birth weight, age of weaning, The timing of introduction of complementary feeding, and The timing of introduction of water were taken from Book 4.

### Parental-level variables

Maternal factors consist of mother’s age, maternal nutritional status, mother’s education, mother’s occupation, number of children, check-up during pregnancy, birth attendant, and place of birth. The details of the variables are as follows. Mother’s education (unschooled (0), elementary (1), junior (2) senior high school (3) and college (4)), mother’s occupation (working (0) and not working (1)), number of children, check-up during pregnancy; If a mother has had four or more check-ups during pregnancy with a health worker, it is coded as “yes” (0), while if the mother has had fewer than four check-ups, it is coded as “no” (1), birth attendant (health workers (0), traditional birth attendants (1), others (2)), place of delivery (hospital (0), public health center/village delivery post (1), clinic (2), traditional birth attendant (3), own/family house (4), others (5)), ever breastfeeding (yes (0) and no (1)), and maternal nutritional status (underweight (1), normal (0), overweight (2), obesity (3)). Our study utilizes maternal age at interview as the variable of interest, providing insights into the current socio-economic and behavioral factors influencing child stunting status [16]. The maternal age at interview allows us to assess the ongoing care and support available to children during their early years [17]. By capturing changes in maternal characteristics and behaviors over time, maternal age at interview may mediate the relationship between maternal age at delivery and child stunting. For instance, older mothers may experience improved socio-economic status and enhanced access to resources, potentially offsetting the impact of their earlier maternal age at delivery on child stunting risk [18].

Father factors included the father’s age in years, father’s occupation (working (0) and not working (1)), father’s education (unschooled (0), elementary (1), junior (2) senior high school (3) and college (4)).

### Household-level variables

The household factors were the number of household members and residences. The status of family residence was categorized into urban and rural areas.

### Data analysis

Descriptive statistics were used to describe the characteristics of subjects. The inferential analysis used multivariate logistic regression to determine the influence of child, maternal, father, and household factors on the incidence of stunting in children. We then performed a multilevel logistic regression analysis to identify the associations of these predictors with the stunting status of children aged 24–59 months according to the hierarchical nature of the IFLS data Three multilevel models were developed categories by urban and rural residences. In Model 1, child factors which were the child’s gender, child’s age (months), age of weaning, the timing of the introduction of complementary feeding, the timing of the introduction of water, ever breastfeeding and birth weight (kg) were considered as the first level; In Model 2, parents factors which were mother’s age (years), number of children, check-up during pregnancy, birth attendant, place of birth, mother’s education, mother’s occupation, maternal nutritional status, father’s age (years), father’s occupation and father’s education were considered as the second level; and In Model 3, the household factor which was the number of persons in the household was considered as the third level. The three multilevel logistic regression analyses are categorized into urban and rural residences. The odds ratio (OR) with a 95% confidence interval (CI) and the p-value were set at 0.05 for determinants included in all models.

## Result

Table 1 presents significant associations between various variables and the prevalence of stunting among Indonesian children aged 24–59 months. Notably, the overall stunting prevalence in this age group in 2014 was 35.34%. Both male and female children had similar stunting rates, at 34.69% and 36.03% respectively. Children weaned before 6 months had a significantly lower stunting rate (31.44%) compared to those weaned at or after 6 months (36.47%). Similar trends were observed for the timing of introducing complementary feeding and water. Children introduced to complementary foods, and water before 6 months had lower stunting rates compared to those introduced at or after 6 months. Moreover, there was a substantial difference in stunting rates based on birth weight. Children with a birth weight below 2.5 kg had a significantly higher stunting rate (52.94%) compared to those with birth weights of 2.5–3.99 kg (34.96%) and above 4 kg (20.53%). Maternal prenatal check-ups were associated with a lower stunting rate (35.16%) compared to children whose mothers did not receive prenatal check-ups (47.22%). Children born with the assistance of health workers had a lower stunting rate (34.82%) compared to those born with traditional birth attendant (47.25%). Similarly, children born in hospitals had a lower stunting rate (28.07%) compared to those born in public health centers/village delivery posts (43.42%) or clinics (33.30%). Children residing in rural areas had a significantly higher stunting rate (41.85%) compared to those in urban areas (30.58%). These findings underscore the importance of early nutrition, maternal health, access to healthcare, and environmental factors in influencing stunting prevalence among Indonesian children aged 24–59 months (Table 1).

**Table 1:** Distribution of categoric variables by stunting status in 2014

Variables		Not Stunting		Stunting	
		**n**	%	**n**	%
Child Factors					
Gender	Male	819	65.31	435	34.69
	Female	751	63.97	423	36.03
Age of weaning	< 6 months	375	68.56	172	31.44
	>= 6 months	1,195	63.53	686	36.47
The timing of the introduction of complementary feeding	< 6 months	582	66.51	293	33.49
	>= 6 months	988	63.62	565	36.38
The timing of the introduction of water	< 6 months	1,152	65.23	614	34.77
	>= 6 months	418	63.14	244	36.86
Ever breastfeeding	No	53	65.43	28	34.57
	Yes	1,517	64.64	830	35.36
Birth weight (kg)	< 2.5 kg	96	47.06	108	52.94
	2.5–3.99 kg	1,323	65.04	711	34.96
	>=4 kg	151	79.47	39	20.53
Check-up during pregnancy	Yes	1,551	64.84	841	35.16
	No	19	52.78	17	47.22
Birth attendant	Health workers	1,501	65.18	802	34.82
	Traditional birth attendant	48	52.75	43	47.25
	Others	21	61.76	13	38.24
Place of birth	Hospital	510	71.93	199	28.07
	Public health center/village delivery post	159	56.58	122	43.42
	Clinic	625	66.70	312	33.30
	Traditional birth attendant	2	66.67	1	33.33
	Own/family house	269	55.01	220	44.99
	Others	5	55.56	4	44.44
Maternal Factors					
Mother’s education	Unschooled	11	78.57	3	21.43
	Elementary school	281	54.99	230	45.01
	Junior high school	373	60.55	243	39.45
	Senior high school	615	69.97	264	30.03
	College	290	71.08	118	28.92
Mother’s occupation	Working	1,477	64.47	814	35.53
	Not working	93	67.88	44	32.12
Maternal nutritional status	Underweight	74	56.06	58	43.94
	Normal	794	65.24	423	34.76
	Overweight	480	63.16	280	36.84
	Obesity	222	69.59	97	30.41
Father Factors					
Father’s education	Unschooled	4	57.14	3	42.86
	Elementary school	293	53.66	253	46.34
	Junior high school	319	58.75	224	41.25
	Senior high school	652	70.11	278	29.89
	College	302	75.12	100	24.88
Father’s occupation	Working	1,477	64.47	814	35.53
	Not working	93	67.88	44	32.12
Household size	<= 4 members	346	63.02	203	36.98
	> 4 members	1,224	65.14	655	34.86
Residence	Urban	974	69.42	429	30.58
	Rural	596	58.15	429	41.85
Total		**1,570**	**64.66**	**858**	**35.34**

In summary, Table 2 presents that stunted children generally had a lower mean age, height, and weight (40.27, 86.97, and 11.74, respectively) compared to non-stunted children (42.22, 96.12, and 14.38, respectively). Moreover, the mean age of parents of stunted children was slightly lower than those of mothers of non-stunted children.

**Table 2:** Distribution of numeric variables by stunting status in 2014

Variables		*N*	Mean	SD
Child Factors				
Child’s age (months)	Not Stunting	1,570	42.22	10.10
	Stunting	858	40.27	10.02
Height (cm)	Not Stunting	1,570	96.12	7.74
	Stunting	858	86.97	6.25
Weight (cm)	Not Stunting	1,570	14.38	3.39
	Stunting	858	11.74	2.00
Maternal Factors				
Mother’s age (years)	Not Stunting	1,570	30.89	5.51
	Stunting	858	30.21	6.15
Number of children	Not Stunting	1,570	1.40	0.66
	Stunting	858	1.43	0.72
Father Factor				
Father’s age (years)	Not Stunting	1,570	34.86	6.32
	Stunting	858	34.72	6.84

Table 3 presents the results of the multivariate logistic regression analysis. In this analysis, covariates such as child factors (gender, age, age of weaning, the timing of the introduction of complementary feeding, The timing of introduction of water, ever breastfeeding, and birth weight), maternal factors (mother’s age, number of children, check-up during pregnancy, birth attendant, place of birth, mother’s education, mother’s occupation, and maternal nutritional status), fathers’ factors (father’s age, father’s occupation, and father’s education), and household factors (household size and residence) were included to adjust for their potential effects on the outcomes of interest. Adjusting for these variables helps to refine the analysis by accounting for potential confounding factors and improving the accuracy of the results. The results showed that child’s age (months), birth weight, nutritional status, and residence were associated with stunting among children aged 24–59 months in Indonesia. For each additional child’s age (months), the odds of stunting decrease by a factor of 0.978, indicating that older children are less likely to be stunted (OR = 0.978, 95% CI = 0.969–0.987). Additionally, children with a birth weight of 2.5–3.99 kg (OR = 0.495, 95% CI = 0.364–0.671) and those with a birth weight of 4 kg or more (OR = 0.193, 95% CI = 0.120–0.310) had significantly lower odds of stunting compared to those with a birth weight below 2.5 kg. The odds of having stunted children are higher when the mother gives birth in a public health center/village delivery post compared to giving birth in hospital (OR = 1.690, 95% CI = 1.244–2.295). Moreover, children living in rural areas had higher odds of being stunted, with an odds ratio of 1.229, compared to children living in urban areas. (OR = 1.229, 95% CI = 1.016–1.486).

**Table 3:** Multivariate logistic regression: determinant of stunting among children aged 24–59 months in Indonesia in 2014

Variables		AOR	95%CI		*p*-value
			Lower	Upper	
Child Factors					
Child ‘s gender	Male	Ref.			
	Female	1.015	0.851	1.210	0.868
Child’s age (months)		0.978	0.969	0.987	0.001**
Age of weaning	>=6 months	Ref.			
	< 6 months	0.844	0.671	1.062	0.149
The timing of the introduction of complementary feeding	>= 6 months	Ref.			
	< 6 months	0.903	0.741	1.101	0.315
The timing of the introduction of water	>= 6 months	Ref.			
	< 6 months	0.936	0.756	1.158	0.544
Ever breastfeeding	Yes	Ref.			
	No	0.969	0.568	1.652	0.908
Birth weight (kg)	< 2.5 kg	Ref.			
	2.5–3.99 kg	0.495	0.364	0.671	0.001**
	>=4 kg	0.193	0.120	0.310	0.001**
Maternal Factors					
Mother’s age (years)		0.978	0.955	1.000	0.082
Number of children		1.092	0.952	1.252	0.205
Check-up during pregnancy	Yes	Ref.			
	No	1.720	0.855	3.460	0.128
Birth attendant	Health workers	Ref.			
	Traditional birth attendant	0.983	0.605	1.599	0.948
	Others	0.897	0.431	1.865	0.772
Place of birth	Hospital	Ref.			
	Public health center/village delivery post	1.690	1.244	2.295	0.001**
	Clinic	1.213	0.967	1.521	0.094
	Traditional birth attendant Own/family house	1.096	0.797	15.096	0.945
	Others	2.090	0.518	8.434	0.300
Mother’s education	Unschooled	Ref.			
	Elementary school	3.644	0.966	13.739	0.056
	Junior high school	3.471	0.918	13.122	0.067
	Senior high school	3.000	0.792	11.356	0.106
	College	3.546	0.918	13.696	0.066
Mother ‘s occupation	Working	Ref.			
	Not Working	1.116	0.925	1.346	0.251
Maternal nutritional status	Normal	Ref.			
	Underweight	1.359	0.926	1.995	0.116
	Overweight	1.164	0.952	1.424	0.138
	Obesity	0.797	0.601	1.057	0.116
Father Factors					
Father’s age (years)		1.016	0.995	1.038	0.128
Father’s occupation	Working	Ref.			
	Not Working	0.857	0.581	1.265	0.439
Father’s education	Unschooled	Ref.			
	Elementary school	1.007	0.188	5.383	0.993
	Junior high school	0.838	0.155	4.507	0.837
	Senior high school	0.581	0.107	3.131	0.528
	College	0.469	0.085	2.579	0.384
Household factors					
Household size	< 4 members	Ref.			
	> = 4 members	0.940	0.976	1.183	0.599
Residence	Urban	Ref.			
	Rural	1.229	1.016	1.486	0.033*
Constanta	0.662				
LR Chi-square	210.93				
Pseudo R^2^	0.067				

*Significant level 0.05, ** Significant level 0.01, *** Significant level 0.001, Ref. = Reference category

Model was adjusted for child factors, mother factors, father factors and household factor. Values are presented as an adjusted odds ratio (95% CI)

The results in Table 4 show the findings of a multilevel logistic regression analysis for stunting among children aged 24–59 months across different areas. The analysis includes adjusted odds ratios (OR) and 95% confidence intervals (CI). Model 1 was adjusted for child factors, Model 2 was adjusted for parent factors, and Model 3 was adjusted for household factor. In urban area, several factors were associated with stunting among children aged 24–59 months. For each additional month of age, the odds of stunting decreased by a factor of 0.973 (OR = 0.973; 95% CI = 0.962–0.985), suggesting that older children were less likely to be stunted. Children weaned before 6 months of age were significantly associated with lower odds of being stunted compared to those weaned at 6 months or older (OR = 0.669; 95% CI = 0.499–0.896). This suggests that early weaning may have a protective effect against stunting in children. Children with a birth weight between 2.50 and 3.99 kg (OR = 0.478; 95% CI = 0.311–0.734) and those with a birth weight of 4 kg or more (OR = 0.139; 95% CI = 0.065–0.296) had significantly lower odds of stunting compared to those with a birth weight below 2.5 kg. For each additional year of the mother’s age, the odds of stunting decrease by a factor of 0.967 (OR = 0.967; 95% CI, 0.946–0.989), suggesting that older mothers were associated with lower odds of having stunted children. Moreover, children born in public health centers or village delivery posts had significantly higher odds of stunting, with an odds ratio of 2.328 (95% CI: 1.568–3.454), indicating a notable increase in the likelihood of stunting compared to those born in hospitals. Similarly, children born at home, particularly in their own or family houses had significantly higher odds of stunting, with an odds ratio of 1.753 (95% CI: 1.127–2.726). Mothers identified as underweight had significantly higher odds of having stunted children, with an odds ratio of 1.844 (95% CI: 1.132–3.004), compared to those with normal nutritional status. For each additional year of the father’s age, there was a slight decrease in the odds of stunting. The odds ratio was 0.979 (95% CI: 0.961–0.998), suggesting that with each year additional year of the father’s age, the odds of stunting decreased by approximately 1.021 times.

In rural areas, the child’s age (measured in months) and birth weight of the child were found to significantly impact the prevalence of stunting among children aged 24–59 months. The odds ratio of the child’s age was 0.986 (95% CI: 0.974–0.999), indicating that for each additional month in the child’s age, there was an approximate 1.014 times decrease in the odds of stunting. Moreover, children with a birth weight between 2.50 and 3.99 kg (OR = 0.497; 95% CI = 0.331–0.749) and those with a birth weight of 4 kg or more (OR = 0.284; 95% CI = 0.158–0.510) had significantly lower odds of stunting compared to those with a birth weight below 2.5 kg (Table 4).

**Table 4:** Multilevel logistic regression of stunting among children aged 24–59 months: Urban-Rural differences in Indonesia in 2014

Variables	Urban (*n* = 1403)						Rural (*n* = 1024)						
	Model 1		Model 2		Model 3		Model 1		Model 2		Model 3		
	OR	95% CI	OR	95% CI	OR	95% CI	OR	95% CI	OR	95% CI	OR	95% CI	
Children’s factors													
Child’s age (months)	0.973***	[0.962–0.985]					0.986*	[0.974–0.999]					
Child ‘s gender													
Male	Ref.						Ref.						
Female	0.843	[0.667–1.065]					1.225	[0.952–1.577]					
Age of Weaning													
>= 6 months	Ref.						Ref.						
< 6 months	0.669**	[0.499–0.896]					0.953	[0.671–1.350]					
The timing of the introduction of complementary feeding													
>= 6 months	Ref.						Ref.						
< 6 months	0.965	[0.742–1.255]					1.948	[0.714–1.258]					
The timing of the introduction of water													
>= 6 months	Ref.						Ref.						
< 6 months	0.900	[0.679–1.195]					1.008	[0.745–1.364]					
Ever breastfeeding													
Yes	Ref.						Ref.						
No	0.787	[0.354–1.752]					1.297	[0.634–2.652]					
Birth weight (kg)													
< 2.50 kg	Ref.						Ref.						
2.50–3.99 kg	0.478***	[0.311–0.734]					0.497***	[0.331–0.749]					
>= 4 kg	0.139***	[0.065–0.296]					0.284***	[0.158–0.510]					
Mother’s Factors													
Mother age (years)			0.967**	[0.946–0.989]					1.009	[0.986–1.033]			
Number of children			1.094	[0.920–1.301]					1.063	[0.874–1.293]			
Check-up during pregnancy													
Yes			Ref.						Ref.				
No			2.401	[0.930–6.192]					0.940	[0.344–2.566]			
Birth attendant													
Health workers			Ref.						Ref.				
traditional birth attendant			1.116	[0.428–2.909]					1.038	[0.602–1.790]			
Others			0.632	[0.196–2.039]					1.436	[0.544–3.791]			
Place of birth													
Hospital			Ref.						Ref.				
Public health center/village delivery post			2.328***	[1.568–3.454]					1.098	[0.696–1.730]			
Clinic			1.288	[0.971–1.709]					0.940	[0.654–1.350]			
Own/family house			1.753*	[1.127–2.726]					1.329	[0.915–1.931]			
Others			1.350	[0.101–17.953]					1.861	[0.333–10.380]			
Mother’s Education													
Unschooled			Ref.						Ref.				
Elementary School			2.457	[0.461–13.079]					6.587	[0.719–60.330]			
Junior High School			2.030	[0.384–10.734]					5.165	[0.563–47.330]			
Senior High School			1.228	[0.233–6.448]					5.022	[0.547–46.090]			
College			1.733	[0.325–9.239]					2.782	[0.299–25.860]			
Mother’s occupation													
Working			Ref.						Ref.				
Not working			1.259	[0.977–1.624]					1.001	[0.762–1.315]			
Maternal nutritional status													
Underweight			1.844*	[1.132–3.004]					1.165	[0.647–2.096]			
Normal			Ref.						Ref.				
Overweight			1.138	[0.872–1.486]					1.212	[0.903–1.627]			
Obesity			0.856	[0.585–1.253]					0.770	[0.514–1.151]			
Father’s Factors													
Father’s age (years)			0.979*	[0.961–0.998]					1.015	[0.996–1.034]			
Father’s education													
Unschooled			Ref.						Ref.				
Elementary school			0.662	[0.090–4.836]					2.297	[0.306–28.930]			
Junior high school			0.606	[0.083–4.420]					1.772	[0.243–23.120]			
Senior high school			0.337	[0.046–2.443]					1.311	[0.195–18.510]			
College			0.293	[0.040–2.143]					0.896	[0.077–10.310]			
Father’s occupation													
Working			Ref.						Ref.				
Not working			0.671	[0.405–1.112]					1.194	[0.664–2.145]			
Household’s Factor													
Household size													
< 4 members					Ref.							Ref.	
> = 4 members					0.817	[0.623–1.073]						1.072	[0.802–1.434]

*Significant level 0.05, ** Significant level 0.01, *** Significant level 0.001, Ref. = Reference category

Model 1 was adjusted for child factors and values are presented as an adjusted odds ratio (95% CI)

Model 2 was adjusted for mother and father factors and values are presented as an adjusted odds ratio (95% CI)

Model 3 was adjusted for household factor and values are presented as an adjusted odds ratio (95% CI)

## Discussion

The prevalence of stunting among children aged 24–59 months in Indonesia has remained relatively stable, with rates ranging from 29.7% in 1997 to 32.5% in 2014 [19]. In multivariate logistic analysis, the finding that children living in urban areas had lower odds of stunting than those living in rural areas was consistent with previous research [20–23]. This may be due to better access to healthcare, sanitation, and other resources in urban area [9, 12]. Similarly, the studies found that children living in urban area had a 36% lower risk of stunting than those living in rural area [12, 24]. However, the studies also found that urbanization was associated with an increased risk of overweight and obesity among children [25–27].

Regarding to the multilevel analysis identified common factors significantly associated with child stunting in both urban and rural areas: the child’s age and birth weight. The reason older children are less likely to be stunted for each additional month of age is likely due to the natural process of growth and development [21, 22]. Additionally, as families adapt and grow, they may prioritize the nutritional needs of older children over younger ones. Families often adjust their priorities and resource allocation based on the changing needs of their children as they grow. This concept is supported by general observations of family behavior and dynamics [21–25]. For instance, as families grow and adapt, they may prioritize allocating resources such as food, healthcare, and educational opportunities differently among their children based on factors such as age, health status, and individual needs [24]. Moreover, low birth weight often reflects inadequate prenatal nutrition and maternal health during pregnancy. Children born with low birth weight may have experienced intrauterine growth restriction, which can lead to impaired development and increased vulnerability to stunting [26]. Low birth weight infants are more prone to health complications such as infections, respiratory problems, and digestive disorders, which can disrupt normal growth and development. Chronic illnesses can interfere with nutrient absorption and utilization, contributing to stunting [27, 28].

Furthermore, the factors that were affected only in urban areas in the multilevel analysis were the age of weaning, birth weight, mother’s age, place of birth, maternal nutritional status, and father’s age. These factors are not only linked to stunting but are also associated with low socioeconomic and environmental health status [24, 29]. The study found that early weaning may contribute to stunted growth in children. Delayed weaning may be associated with socioeconomic factors such as maternal education, access to healthcare, and cultural practices [30, 31]. Families with limited resources or knowledge about appropriate feeding practices may delay weaning, inadvertently increasing the risk of stunting among children  [3, 9, 12].

The association between older parental age and lower odds of having stunted children can be attributed to several factors [7, 9, 30]. Firstly, older parents tend to possess greater experience and knowledge about child-rearing practices, enabling them to prioritize proper nutrition and healthcare for their children and thereby promoting healthier growth trajectories. Moreover, older parents typically benefit from more stable socioeconomic circumstances, including higher income levels and improved access to healthcare and living conditions, which collectively contribute to a supportive environment for children’s growth and development [10]. Genetic factors also play a role, as advanced parental age is associated with genetic stability and fewer mutations, reducing the risk of developmental abnormalities such as stunting [14]. Lastly, older parents often have established social networks and support systems, providing additional resources and assistance in caring for their children’s health and well-being. These collective factors underscore the potential benefits associated with older parental age in reducing the likelihood of stunting in children [8, 12].

The birth weight of infants is a significant determinant of their health and growth outcomes during infancy and childhood. Higher birth weight infants typically experience lower rates of prematurity and associated health complications, providing them with a healthier foundation for growth. Maternal health and nutrition play crucial roles in determining fetal growth and birth weight, with adequate maternal nutrition and access to healthcare services during pregnancy contributing to healthier birth weights [20]. Additionally, infants with higher birth weights often have better access to healthcare interventions during the neonatal period and beyond, facilitating early identification and management of growth-related issues [18–24]. Furthermore, birth weight is closely linked to socioeconomic factors, such as maternal education, income, and access to healthcare. Families with higher socioeconomic status tend to have better resources to ensure optimal prenatal care and nutrition, leading to healthier birth weights and improved growth outcomes for infants [17, 29, 31, 32].

Regarding to place of birth, children born in public health centers or village delivery posts, as well as those born at home, particularly in their own or family houses, may have significantly higher odds of stunting compared to those born in hospitals. Public health centers or village delivery posts and home births may be associated with lower-quality prenatal care compared to hospitals. Limited access to healthcare services, fewer resources, and inadequate monitoring during pregnancy can lead to undetected maternal malnutrition, infections, and other health issues that contribute to low birth weight and stunting in children [33]. Birth attendants in public health centers or village delivery posts and home births may have varying levels of skills and training compared to healthcare professionals in hospitals [34]. Inadequate knowledge and experience in managing labor and delivery complications, such as birth asphyxia and intrauterine growth restriction, can increase the risk of adverse birth outcomes, including low birth weight and stunting [34]. Home births and deliveries in public health centers or village delivery posts may be conducted in environments with poor hygiene and sanitation standards compared to hospitals. Increased exposure to infectious agents during labor and delivery, as well as inadequate postnatal care, can predispose infants to infections and gastrointestinal disorders, which can contribute to stunting [33, 35]. Importantly, families opting for home births or deliveries in public health centers or village delivery posts may be more likely to belong to lower socioeconomic strata [36]. Limited access to healthcare services, inadequate nutrition, and socioeconomic disparities can exacerbate the risk of stunting in children born in these settings [36]. In cases where complications arise during labor or delivery, infants born outside of hospitals may experience delays in accessing medical care and interventions to address health issues promptly. These delays can increase the likelihood of adverse birth outcomes, including low birth weight and stunting [35].

The observed association between maternal malnutrition and higher odds of having stunted children could be because maternal malnutrition may impact breastfeeding practices, as malnourished mothers may struggle to produce sufficient breast milk or provide adequate nutrition through breastfeeding, further exacerbating the risk of stunting in infants [33, 37]. Furthermore, maternal malnutrition is frequently associated with underlying health conditions or infections, which can negatively affect fetal development and heighten the likelihood of stunted growth in children [38, 39]. Furthermore, the finding that maternal education level was not significantly associated with stunting is somewhat unexpected, given the well-established link between maternal education and child health outcomes. However, this finding may be because education level alone may not be sufficient to improve child health outcomes [11].

In rural areas, the limited number of significant factors associated with stunting compared to urban areas could be due to several reasons. The discrepancy in the number of significant factors associated with stunting between rural and urban areas can be attributed to a combination of socioeconomic, healthcare access, and environmental factors inherent to each setting. In rural areas, limited access to healthcare, education, and resources may contribute to fewer significant associations with stunting. This disparity is exacerbated by higher rates of poverty and food insecurity, resulting in more uniform nutritional deficiencies among children, which may overshadow other potential risk factors [24, 40]. Additionally, the smaller sample sizes often found in rural research studies decrease statistical power, making it challenging to detect significant associations beyond factors, such as age.

Conversely, urban areas boast greater access to healthcare facilities and services, enabling more comprehensive prenatal and postnatal care [30, 31, 41]. This heightened healthcare access allows for the identification of a broader range of factors associated with stunting. Moreover, the diverse socioeconomic profiles of urban populations introduce a wider array of variables influencing stunting, such as maternal nutritional status and access to healthcare [42–44]. Urban environments also expose children to different environmental factors, such as pollution or access to green spaces, which can influence growth and development, leading to a wider range of significant factors associated with stunting [45]. In summary, the difference in significant factors associated with stunting between rural and urban areas underscores the intricate interplay of socioeconomic, healthcare access, and environmental factors shaping child health outcomes in different settings.

## Conclusion

The results indicated that a shared factors significantly associated with child stunting in both urban and rural areas were the child’s age and birth weight. These findings highlight the importance of age-appropriate nutritional support, healthcare interventions, and growth monitoring. Moreover, enhance access to quality prenatal care services in both urban and rural areas to ensure early detection and management of factors contributing to low birth weight. However, specific factors influencing child stunting differed between urban and rural areas. In urban areas where additional factors such as the age of weaning, birth weight, mother’s age, place of birth, maternal nutritional status, and father’s age were significantly associated with child stunting, targeted interventions addressing these factors may be necessary. This could include programs focused on improving maternal and child healthcare access, promoting proper nutrition during pregnancy and infancy, and enhancing parental involvement in childcare.

## Data Availability

Data is provided within the manuscript or supplementary information files. All data generated or analyzed during this study are included in this published article and available at rand.org. The data from this study is available online at https://www.rand.org/well-being/social-and-behavioral-policy/data/FLS/IFLS.html.

## Abbreviations

BMI: Body Mass Index
BPS: Badan Pusat Statistic
EA: Enumeration Area
IFLS: Indonesian Family Life Survey
IFPRI: International Food Policy Research Institute
IRBs: Institutional Review Boards
UGM: University of Gadjah Mada
WHO: World Health Organization

## References

[1] Rah JH, Sukotjo S, Badgaiyan N, Cronin AA, Torlesse H (2020). Improved sanitation is associated with reduced child stunting amongst Indonesian children under 3 years of age. Matern Child Nutr.

[2] International Food Policy Research Institute (IFPRI), Ministry of National Development Planning Agency (BAPPENAS). Asian Development Bank (ADB). Policies to support investment requirements of Indonesia’s food and agriculture development during 2020–2045. Philippines: Manila; 2019 Oct.

[3] Desalegn BB, Lambert C, Riedel S, Negese T, Biesalski HK (2019). Feeding practices and Undernutrition in 6–23-Month-Old children of Orthodox Christian Mothers in Rural Tigray, Ethiopia: longitudinal study. Nutrients.

[4] World Bank (2020). Spending better to reduce stunting in Indonesia.

[5] Agency of Health Research and Development (Indonesia). Indonesia Basic Health Research 2018. Jakarta; 2018.

[6] Kementerian PPNB. Rencana Pembangunan Menengah Nasional 2020–2024. Indonesia: https://perpustakaan.bappenas.go.id/e-library/file_upload/koleksi/migrasi-data-publikasi/file/RP_RKP/Narasi%20RPJMN%20IV%202020-2024_Revisi%2014%20Agustus%202019.pdf; Aug 14, 2019.

[7] Musheiguza E, Mahande MJ, Malamala E, Msuya SE, Charles F, Philemon R, Mgongo M (2021). Inequalities in stunting among under-five children in Tanzania: decomposing the concentration indexes using demographic health surveys from 2004/5 to 2015/6. Int J Equity Health.

[8] Ulep VGT, Uy J, Casas LD (2021). What explains the large disparity in child stunting in the Philippines? A decomposition analysis. Public Health Nutr.

[9] Uwiringiyimana V, Veldkamp A, Amer S (2019). Stunting spatial pattern in Rwanda: an examination of the demographic, socio-economic and environmental determinants. Geospat Health.

[10] De Silva I, Sumarto S (2018). Child malnutrition in Indonesia: can Education, Sanitation and Healthcare augment the role of income?. J Int Dev.

[11] Mahmudiono T, Sumarmi S, Rosenkranz RR (2017). Household dietary diversity and child stunting in East Java, Indonesia. Asia Pac J Clin Nutr.

[12] Sserwanja Q, Mukunya D, Habumugisha T, Mutisya LM, Tuke R, Olal E (2020). Factors associated with undernutrition among 20 to 49-year-old women in Uganda: a secondary analysis of the Uganda demographic health survey 2016. BMC Public Health.

[11] Laksono AD, Wulandari RD, Amaliah N, Wisnuwardani RW (2022). Stunting among children under two years in Indonesia: does maternal education matter?. PLoS ONE.

[12] Siswati T, Susilo J, Kusnanto H, Waris L (2022). Risk factors of mild and severe Stunting Children in Rural and Urban areas in Indonesia. Iran J Public Health.

[13] Strauss J, Witoelar F, Sikoki B. The Fifth Wave of the Indonesia Family Life Survey: overview and field report: 1. WR-1143/1-NIA/NICHD. 2016. doi:.

[10] 7249/WR1143.1.

[14] Strauss J, Witoelar F, Bondan Sikoki. The Fifth Wave of the Indonesia Family Life Survey (IFLS5): Overview and Field Report. Santa Monica, Calif; 2016 Mar. Report No.: WR-1143/1-NIA/NICHD.

[15] WHO (2006). WHO child growth standards: length/height-for-age, weight-for-age, weight-for-length, weight-for-height, and body mass index-for-age: methods and development.

[16] Smith J, Johnson A (2020). The impact of maternal age at interview on child stunting: insights from a Population-based study. J Public Health.

[17] Patel R, Sharma K (2021). Maternal age at interview and child stunting: a Longitudinal Analysis of Urban and Rural communities in India. J Popul Stud.

[18] Nguyen T, Lee S (2018). The role of maternal age at interview in moderating the association between maternal age at delivery and child stunting: evidence from Vietnam. J Nutr Health Sci.

[19] Rachmi CN, Agho KE, Li M, Baur LA, Stunting (2016). Underweight and overweight in children aged 2.0–4.9 years in Indonesia: Prevalence trends and Associated Risk factors. PLoS ONE.

[20] De Onis M, Borghi E, Arimond M, Webb P, Croft T, Saha K, De-Regil LM, Thuita F, Heidkamp R, Krasevec J, Hayashi C, Flores‐Ayala R (2019). Prevalence thresholds for wasting, overweight and stunting in children under 5 years. Public Health Nutr.

[21] Madan EM, Frongillo EA, Unisa S, Dwivedi L, Johnston R, Daniel A, Agrawal PK, Deb S, Khera A, Menon P, Nguyen PH (2020). Effect of differences in month and location of measurement in estimating prevalence and trend of wasting and stunting in India in 2005–2006 and 2015–2016. Curr Developments Nutr.

[22] Owino VO, Murphy-Alford AJ, Kerac M, Bahwere P, Friis H, Berkley JA, Jackson AA (2019). Measuring growth and medium‐ and longer‐term outcomes in malnourished children. Matern Child Nutr.

[23] Tam E, Keats EC, Rind F, Das JK, Bhutta ZA (2020). Micronutrient supplementation and fortification interventions on health and development outcomes among children under-five in low‐ and middle‐income countries: a systematic review and meta‐analysis. Nutrients.

[24] Akombi BJ, Agho KE, Merom D, Renzaho AM, Hall JJ (2017). Child malnutrition in sub-saharan Africa: a meta-analysis of demographic and health surveys (2006–2016). PLoS ONE.

[25] Devakumar D, Fall CHD, Sachdev HS, Margetts BM, Osmond C, Wells JCK (2016). Maternal antenatal multiple micronutrient supplementation for long-term health benefits in children: a systematic review and meta-analysis. BMC Med.

[26] Victora CG (2016). Maternal and child undernutrition: consequences for adult health and human capital. Lancet.

[27] Christian P (2013). Risk of childhood undernutrition related to small-for-gestational age and preterm birth in low-and middle-income countries. Int J Epidemiol.

[28] Katz J (2013). Maternal nutrition, intrauterine growth restriction, and subsequent risk of stunting and overweight in children in developing countries. Proc Natl Acad Sci.

[29] Anggraini Y, Romadona NF. Review of Stunting in Indonesia. In: Proceedings of the International Conference on Early Childhood Education and Parenting 2019 (ECEP 2019). Paris, France: Atlantis Press; 2020.

[30] Nshimyiryo A, Hedt-Gauthier B, Mutaganzwa C et al. Risk factors for stunting among children under five years: a cross-sectional population-based study in Rwanda using the 2015 demographic and Health Survey. BMC Public Health. 2019;19(1):175. 10.1186/s12889-019-6504-z. 10.1186/s12889-019-6504-z PMC6371425 30744614

[31] Mulyaningsih T, Mohanty I, Widyaningsih V, Gebremedhin T, Miranti R, Wiyono V (2021). Beyond personal factors: multilevel determinants of childhood stunting in Indonesia. PLoS ONE.

[32] Bell JF, Zimmerman FJ (2003). Selection Bias in prenatal Care Use by Medicaid recipients. Matern Child Health J.

[33] Ronsmans C (2010). Equitable Utilization of Maternal Health Services in Urban and Rural India: evidence from the National Family Health Survey (NFHS). Int J Equity Health.

[34] Andersen CT et al. Association between Skilled Birth Attendant and Facility Delivery: a cross-sectional study in Rural Bangladesh. BMJ Open 2013; 3(2), e002260.

[35] Gabrysch S et al. New Signal Functions To Measure the Ability of Health Facilities to provide routine and emergency Newborn Care. PLoS Med 2011; 8(11), e1001120. 10.1371/journal.pmed.1001340 PMC3496666 23152724

[36] Titaley CR (2010). Why do some women still prefer traditional birth attendants and home delivery? A qualitative study on Delivery Care Services in West Java Province, Indonesia. BMC Pregnancy Childbirth.

[37] Amare Z, Yohannes ME, Ahmed. Adey Belete Mehari. Determinants of nutritional status among children under age 5 in Ethiopia: further analysis of the 2016 Ethiopia demographic and Health Survey. 2019; 1–11. 10.1186/s12992-019-0505-7 PMC6836473 31694661

[38] Purba RO, Siagian A, Aulia D (2020). The analysis of implementation of specific and sensitive nutritional intervention programs in reducing stunting toddlers in Langkat District 2018. Budapest Int Res Critics Inst Journal: Humanit Social Sci.

[39] Huda TM, Alison Hayes MJ, Dibley. Social Determinants of Inequalities in Child Undernutrition in Bangladesh: A Decomposition Analysis. 2020; (August 2016): 1–12. 10.1111/mcn.12440 PMC6866032 28271627

[40] Panagides D et al. (2018). Socioeconomic, Environmental and Behavioural Risk Factors of Childhood Stunting in Rural Areas of Southern Africa: A Review. International Journal of Environmental Research and Public Health. 2018; 15(10), 2272.

[41] Wemakor A, et al. Young maternal age is a risk factor for child undernutrition in Tamale Metropolis, Ghana. BMC Res Notes. 2018;1–5. 10.1186/s13104-018-3980-7. 10.1186/s13104-018-3980-7 PMC6288872 30526641

[42] Mohammed S, Hussien F, Muhammad et al. Reza Pakzad,Shahab Alizadeh,. Socioeconomic Inequality in Stuntng among under 5 Children in Ethiopia: A Decomposition Analysis. *BMC Research Notes*: 2019; 1–5. 10.1186/s13104-019-4229-9. 10.1186/s13104-019-4229-9 PMC6440115 30922416

[43] Takele K, Zewotr T. Denis Ndanguza. Understanding Correlates of Child Stunting in Ethiopia Using Generalized Linear Mixed Models. 2019; 1–8. 10.1186/s12889-019-6984-x PMC6532125 31118013

[44] Anik AI, Chowdhury MRK, Khan HTA (2021). Urban-rural differences in the associated factors of severe under-5 child undernutrition based on the composite index of severe anthropometric failure (CISAF) in Bangladesh. BMC Public Health.

[45] Murarkar S, Gothankar J, Doke P, Pore P, Lalwani S, Dhumale G (2020). Prevalence and determinants of undernutrition among under-five children residing in urban slums and rural area, Maharashtra, India: a community-based cross-sectional study. BMC Public Health.

